# The Impact of COVID‐19 Pandemic on Prevalence of Diabetic Ketoacidosis at Diagnosis of Type 1 Diabetes: A Single‐Centre Study in Central Pennsylvania

**DOI:** 10.1002/edm2.235

**Published:** 2021-02-17

**Authors:** Kaleb T. Bogale, Valerie Urban, Eric Schaefer, Kanthi Bangalore Krishna

**Affiliations:** ^1^ Penn State College of Medicine Hershey PA USA; ^2^ Division of Pediatric Diabetes and Endocrinology Penn State Hershey Medical Center Hershey PA USA; ^3^ Department of Public Health Sciences Penn State Hershey Medical Center Hershey PA USA

**Keywords:** type 1 diabetes, diabetic ketoacidosis, COVID‐19

## Abstract

**Objective:**

We conducted this study to investigate whether the COVID‐19 pandemic impacted the rate of DKA and previously identified risk factors in children presenting with T1D.

**Methods:**

We performed an extension of a retrospective analysis of all paediatric patients (age ≤ 18) newly diagnosed with T1D within a tertiary care referral centre between 01/01/2017 and 09/14/2020. Demographics, insurance coverage and clinical documents 30 days before their T1D diagnosis were abstracted to assess for symptoms at diagnosis, laboratory values (blood glucose, HbA_1c_, venous pH and bicarbonate) and any healthcare encounters within 30 days of their diagnosis of T1D.

**Results:**

412 patients with T1D [171 F:241 M; 370 pre‐COVID era:42 post‐COVID era] were included. The percentages of DKA diagnoses at admission were very similar between the pre‐COVID and post‐COVID groups (47% vs. 48%), as were the severity (13% vs. 14% mild DKA; 33% vs. 31% moderate or severe DKA).

**Conclusion:**

There were no fluctuations in the rate of DKA among paediatric patients newly diagnosed with T1D throughout the coronavirus pandemic in central Pennsylvania.

## Graphical abstract

We conducted this study to investigate whether the COVID‐19 pandemic impacted the rate of DKA and previously identified risk factors in children presenting with T1D. We performed an extension of a retrospective analysis of all paediatric patients (age ≤ 18) newly diagnosed with type 1 diabetes within a tertiary care referral centre between 01/01/2017 and 09/14/2020. There were no fluctuations in the rate of DKA among paediatric patients newly diagnosed with T1D throughout the coronavirus pandemic in central Pennsylvania.

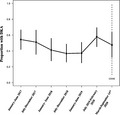

**Abbreviations**
T1D: type 1 diabetes
DKA: diabetic ketoacidosis

## 1 INTRODUCTION

The majority of patients with Type 1 Diabetes Mellitus (T1D) present with similar symptoms including polyuria, polydipsia, weight loss and fatigue. Unfortunately, some patients progress to diabetic ketoacidosis (DKA) at the time of diagnosis of T1D^1^. Children diagnosed in DKA have increased risk of morbidity, mortality, poor glycaemic control, higher medical costs and healthcare resource utilization including ICU level care^1^, ^2^. Previous research has identified the predictors for children to present in DKA, which include younger children (under 5 years of age), Hispanic or African American race, low socioeconomic status, misdiagnosis at an initial clinical encounter and lack of private health insurance^3^. Thus far, there are limited data on the impact of the COVID‐19 pandemic on the rate and severity of DKA in children at initial diagnosis of T1D. Interestingly, some geographic locations noted an increase in DKA frequency during the COVID‐19 pandemic, while others reported no change^4^, ^5^. We conducted this study to investigate whether the COVID‐19 pandemic impacted the rate of DKA and previously identified risk factors in children presenting with T1D in a single tertiary care referral centre in central Pennsylvania.

## 2 METHODS

We performed an extension of a retrospective analysis of all paediatric patients (age ≤ 18) newly diagnosed with T1D within a tertiary care referral centre between 01/01/2017 and 09/14/2020. Demographics, insurance coverage, and all clinical documents 30 days before their T1D diagnosis were abstracted to assess for symptoms at diagnosis (polyuria, polydipsia, nocturia, weight loss, nausea, vomiting, altered mental status, infection, vision changes and autism spectrum disorder), laboratory values (blood glucose, HbA_1c_, venous pH and bicarbonate) and any healthcare encounters within 30 days of their diagnosis of T1D. We performed descriptive statistics and univariate analyses [evaluating children diagnosed with T1D during the pre‐COVID‐19 era (diagnosed between 1/1/2017 and 2/28/2020) and post‐COVID‐19 era (diagnosed between 03/01/2020‐ and 09/14/2020) associated with the incidence of DKA], followed by logistic regression analysis (incorporating key clinical factors previously associated with DKA and the pre‐ or post‐ COVID‐19 era classification). The pH at diagnosis was used to classify DKA as mild DKA (7.25‐<7.30) or moderate/severe DKA (<7.25).

## 3 RESULTS

**FIGURE 1:**
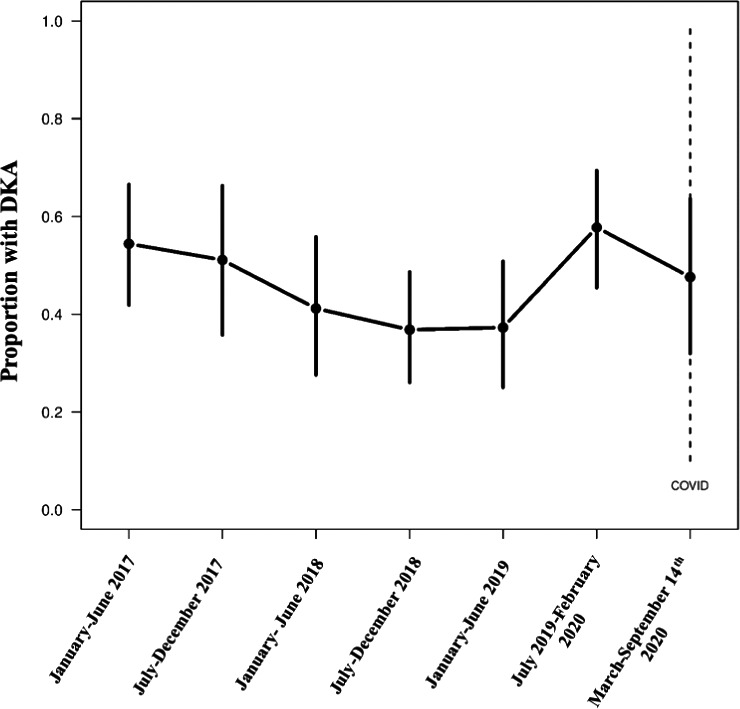
Time series of the proportion of patients with a DKA diagnosis at the time of admission by approximately 6‐month periods from 1 January 2017 to 14 September 2020. The vertical lines for each period represent the 95% confidence interval for the proportion

The analysis included 412 paediatric patients with T1D [171 F:241 M; 370 pre‐COVID‐19 era:42 post‐COVID‐19 era] (TABLE 1). In children diagnosed with T1D in the post‐COVID era, the peak occurrences of T1D were in ages 5‐13 (Interquartile Range [25%‐75%]), males (23, 54.8%), white race (33, 78.6%) and children with Medicaid (16, 38.1%) or military/government (13, 31%) insurance. 8 (19%) children had private insurance and 5 (11.9%) were uninsured (TABLE 1). The rate of DKA was similar between the pre‐COVID‐19 and post‐COVID‐19 groups (47% vs 48%), as was DKA severity (13% vs. 14% mild DKA; 33% vs. 31% moderate or severe DKA). There were no temporal associations with the rate of DKA in respect to COVID‐19 (FIGURE 1); however, age (0‐3 and 9‐13 years), misdiagnosis during a preceding healthcare encounter, presenting to the emergency department directly, elevated HbA_1c_ (>10.0%/13.4mmol/L), and altered mental status (a known symptom of DKA) were associated with increased risk of DKA on multivariable analysis (TABLE 2).

**TABLE 1:** Data summary of the 412 patients [370 Pre‐COVID and 42 Post‐COVID] included in this study

	Pre‐COVID (N = 370)	Post‐COVID (N = 42)
Age		
Mean (Standard deviation)	10.0 (4.29)	9.2 (4.55)
Median	11.0	9.0
Interquartile range (25th, 75th percentile)	7.0, 13.0	5.0, 13.0
Range	(0.0‐18.0)	(1.5‐17.0)
Sex		
Female	152 (41.1%)	19 (45.2%)
Male	218 (58.9%)	23 (54.8%)
Race		
White	261 (70.5%)	33 (78.6%)
Hispanic or Latino	41 (11.1%)	4 (9.5%)
Black or African American	31 (8.4%)	2 (4.8%)
Two or more races	16 (4.3%)	0
Asian	3 (0.8%)	0
Other	11 (3.0%)	3 (7.1%)
Missing	7	0
BMI Percentile		
Mean (standard deviation)	48.9 (34.64)	54.9 (28.94)
Median	48.2	60.8
Interquartile range (25th, 75th percentile)	15.4, 85.3	34.5, 77.1
Range	(0.0‐99.9)	(0.0‐99.9)
Insurance		
Medicaid	137 (37.0%)	16 (38.1%)
Private	206 (55.7%)	8 (19.0%)
Self‐pay	21 (5.7%)	5 (11.9%)
Military	4 (1.1%)	13 (31.0%)
Missing	2	0
Type of Initial Healthcare Encounter		
Primary care provider	266 (71.9%)	27 (64.3%)
Emergency department	104 (28.1%)	15 (35.7%)
Outcome of Primary Care Provider Appointment		
Referred to ED for concern of T1D	216 (58.4%)	24 (57.1%)
Misdiagnosis and discharged without concern of T1D	50 (13.5%)	3 (7.1%)
No PCP visit (went to ED directly)	104 (28.1%)	15 (35.7%)
Language		
English	349 (94.3%)	39 (92.9%)
Other primary language	20 (5.4%)	3 (7.1%)
Missing	1	0
Family History of Type 1 Diabetes		
First degree relative	42 (11.4%)	4 (9.5%)
Second degree relative	73 (19.7%)	9 (21.4%)
No family history	229 (61.9%)	27 (64.3%)
Missing	26	2
Blood Glucose		
Mean (standard deviation)	503.6 (198.41)	486.8 (217.68)
Median	470.0	455.0
Interquartile range (25th, 75th percentile)	378.0, 596.0	358.0, 581.5
Range	(176.0‐1500.0)	(86.0‐1172.0)
Initial A1c		
Mean (standard deviation)	12.0 (2.38)	12.2 (2.47)
Median	11.7	12.7
Interquartile range (25th, 75th percentile)	10.3, 13.9	11.0, 14.0
Range	(6.2‐18.6)	(5.8‐16.5)
Altered Mental Status		
No	349 (94.3%)	40 (95.2%)
Yes	20 (5.4%)	2 (4.8%)
Missing	1	0
Autism		
No	343 (92.7%)	39 (92.9%)
Yes	26 (7.0%)	3 (7.1%)
Missing	1	0
DKA at Diagnosis of T1D		
No	198 (53.5%)	22 (52.4%)
Yes	172 (46.5%)	20 (47.6%)
Severity of DKA at Diagnosis of T1D		
No DKA	198 (53.5%)	22 (52.4%)
Mild DKA	49 (13.2%)	6 (14.3%)
Moderate or severe DKA	123 (33.2%)	13 (31.0%)
Missing	0	1

Abbreviations: ED, emergency department; PCP, primary care provider.

**TABLE 2:** Multivariable logistic regression model of 385 patients in respect to DKA at time of diagnosis of T1D

Parameter	OR (95% CI)	*p*‐value
Age		
0‐3	**4.16 (1.64‐10.6)**	**0.003**
4‐8	1.26 (0.64‐2.48)	0.50
9‐13	**2.21 (1.22‐4.00)**	**0.009**
14‐18 (ref)	1	
Sex		
Male	1.26 (0.78‐2.01)	0.34
Female (ref)	1	
Race		
White	1.70 (0.97‐2.99)	0.07
Other (ref)	1	
BMI percentile		
<15th percentile	1.11 (0.57‐2.17)	0.75
15th to 85th percentile	0.69 (0.39‐1.23)	0.21
>85th percentile (ref)	1	
Insurance		
Private (ref)	1	
Medicaid	1.21 (0.70‐2.07)	0.50
Other	1.00 (0.45‐2.25)	0.99
Outcome of PCP healthcare encounter		
Referred to ED for concern of T1D (ref)	1	
Misdiagnosis and discharged without concern of T1D	**3.73 (1.72‐8.11)**	**0.001**
No PCP Visit (went to ED directly)	**1.76 (1.04‐2.96)**	**0.034**
A1c		
≤10% (13.4mmol/L) (ref)	1	
>10% (13.4mmol/L)	**5.81 (2.96‐11.4)**	**<0.001**
Altered mental status		
Yes	**9.73 (1.21‐78.0)**	**0.032**
No (ref)	1	
Autism		
Yes	0.94 (0.3‐2.34)	0.90
No (ref)	1	
COVID era		
Pre‐COVID (ref)	1	
Post‐COVID	0.94 (0.40‐2.21)	0.89

Note

A total of 27 patients (6.6%) had a missing value for at least one of the variables included in the model. These patients were excluded when fitting the model. Odds ratios (ORs) and corresponding 95% CIs for parameters in the model are shown. Bold font signifies *p*‐value ≤ 0.05.

Abbreviations: ED, emergency department; PCP, primary care provider.

## 4 DISCUSSION

Our study was the first assessment of the rate of DKA at diagnosis of T1D within central Pennsylvania throughout the COVID‐19 pandemic. We found similar overall DKA rates and severity throughout the COVID‐19 pandemic to an earlier assessment of newly diagnosed T1D in the paediatric population in the same region^3^. Alternatively, there are reports of increased frequency^5^ or severity^4^ of DKA throughout the COVID‐19 pandemic in other geographic locations. Regardless, our findings suggest previously described predictors of DKA in the paediatric population persist, even in the setting of the COVID‐19 pandemic^3^. A limitation to this study was the small cohort size in the post‐COVID era. Further investigation of the rate of DKA in other regions of the US during the COVID‐19 pandemic would be of interest, particularly to elucidate any regional differences and their potential causes. The findings of this investigation offer clinicians a simple and cost‐effective means to rapidly risk‐stratify children for development of DKA throughout the COVID‐19 pandemic.

## CONFLICT OF INTEREST

No authors report a conflict of interest.

## AUTHOR CONTRIBUTIONS

K. T. B., V. U. and K. B. K. performed the research and designed the research study. E. S. analysed the data. K. T. B., V. U. and K. B. K. wrote and edited the final manuscript.

## ETHICAL APPROVAL

The study has institutional IRB approval.

## Data Availability

The data that support the findings of this study are available from the corresponding author upon reasonable request.
